# Combined Acupoints for the Treatment of Patients with Obesity: An Association Rule Analysis

**DOI:** 10.1155/2022/7252213

**Published:** 2022-03-17

**Authors:** Ping-Hsun Lu, Yu-Yang Chen, Fu-Ming Tsai, Yuan-Ling Liao, Hui-Fen Huang, Wei-Hsuan Yu, Chan-Yen Kuo

**Affiliations:** ^1^Department of Chinese Medicine, Taipei Tzu Chi Hospital, Buddhist Tzu Chi Medical Foundation, New Taipei City, Taiwan; ^2^School of Post-Baccalaureate Chinese Medicine, Tzu Chi University, Hualien, Taiwan; ^3^Department of Mathematics National Central University, Taoyuan, Taiwan; ^4^Department of Research, Taipei Tzu Chi Hospital, Buddhist Tzu Chi Medical Foundation, New Taipei City, Taiwan

## Abstract

Obesity is a prevalent metabolic disease that increases the risk of other diseases, such as hypertension, diabetes, hyperlipidemia, cardiovascular disease, and certain cancers. A meta-analysis of 11 randomized sham-controlled trials indicates that acupuncture had adjuvant benefits in improving simple obesity, and previous studies have reported that acupoint combinations were more useful than single-acupoint therapy. The Apriori algorithm, a data mining-based analysis that finds potential correlations in datasets, is broadly applied in medicine and business. This study, based on the Apriori algorithm-based association rule analysis, found the association rules of acupoints among 11 randomized controlled trials (RCTs). There were 23 acupoints extracted from 11 RCTs. We used Python to calculate the association between acupoints and disease. We found the top 10 frequency acupoints were Extra12, TF4, LI4, LI11, ST25, ST36, ST44, CO4, CO18, and CO1. We investigated the 1118 association rule and found that {LI4, ST36} ≥ {ST44}, {LI4, ST44} ≥ {ST36}, and {ST36, ST44} ≥ {LI4} were the most associated rules in the data. Acupoints, including LI4, ST36, and ST44, are the core acupoint combinations in the treatment of simple obesity.

## 1. Introduction

Obesity is a disease involving an excessive amount of body fat that increases the risk of conditions, such as cardiovascular diseases, diabetes mellitus, elevated blood pressure, and certain cancers. It is estimated that by the year 2030, there will be 2.16 billion individuals who are overweight and 1.12 billion individuals with obesity globally [1, 2]. Moreover, the obesity and overweight epidemics are not only limited to developed countries but are also increasing among people in developing countries. Obesity is often defined by body mass index (BMI) values. According to the WHO definition, a BMI of over 25 kg/m^2^ indicates an overweight state, while one over 30 kg/m^2^ is considered obese [3]. Simple obesity refers to obesity caused by no obvious etiology of genetic, endocrine, and metabolic diseases, and it is classified as nonpathological obesity [4, 5]. Simple obesity is related to diet habits, lack of exercise, and characteristics of adipose tissue [6–8]. Weight can be managed through dietary changes and lifestyle modifications, such as regular exercise and regular sleeping hours; however, the results may be unsatisfactory [9]. Pharmaceutical treatments, such as fenfluramine and sibutramine, are effective but may be limited due to safety issues [10, 11].

As an alternative intervention, acupuncture is relatively safe and convenient and has been used in clinical practice for obesity therapy [12]. Its possible mechanisms include endocrine system regulation, digestion promotion, oxidative stress reduction, and metabolism modulation [13]. A recent meta-analysis demonstrated that acupuncture therapy is effective for the reduction of BMI, body fat mass (BFM), hip circumference (HC), and waist circumference (WC) [12]. When treating diseases, the Yellow Emperor's Internal Classic and the ancient meridian theory guide the selection and combination of acupoints, which are key for successful acupuncture treatment [14, 15]. However, there is no clear consensus regarding the standard acupoint combinations for simple obesity treatment.

Data mining techniques have recently been used in Chinese medicine and acupuncture. Based on these methods, previous studies provide benchmarks for selecting and combining herbs in the herbal bath for treating uremic pruritus [16], acupoints for treating diabetic gastroparesis [17], chronic obstructive pulmonary disease [18], and Alzheimer's disease [19]. Acupuncture therapy is based on selecting and combining numerous acupoints simultaneously in clinical practice. The Apriori algorithm-based association rule is a promising and useful strategy with which to explore the underlying rules. Nevertheless, there is no consensus on the standard acupoint combinations for obesity treatment. Apriori determines each data's association, identifies the frequency of every item in the database, and finds the association with other objects. This clarifies which items are the most relative [20].

While data mining has been used to explore acupoint combinations for treating obesity, the data they analyzed came from low-level evidence sources, not randomized controlled trials (RCTs), and some were studies of secondary obesity [21, 22]. We explored the promising core acupoint combinations used to treat simple obesity using Apriori association rule analysis via a meta-analysis of 11 RCTs [12].

## 2. Materials and Methods

### 2.1. Data Sources and Selection Criteria

This study was based on a previous meta-analysis of 11 RCTs on acupuncture treatment for obesity. The data on acupoints used for obesity were extracted from 11 RCTs [12]. All enrolled studies were required to meet the following criteria: (1) the patients met the diagnostic criteria for being overweight (23.0 kg/m^2^) for ages >18 set by the Asia-Pacific adult BMI; and (2) standard electro or auricular acupuncture was applied to the experimental group, and sham acupuncture was applied to the control group. The exclusion criteria were as follows: (1) secondary obesity; (2) using other forms of acupuncture; and (3) use of complementary or alternative therapies whose efficacy has not been determined yet in the control group.

### 2.2. Risk of Bias Assessment

We applied the modified Jadad scale to assess the methodological quality of all included studies [23]. This tool contained four domains, including randomization, randomization hidden, and blinding, which were assigned zero to two points, respectively. Withdrawals and dropouts were assigned scores of zero to one. The scores of each domain were combined to evaluate the overall quality of each RCT. A high-quality RCT should get three or more points. The detailed quality scoring was described in a previous study [12]. The Cochrane risk of bias (RoB) 2.0 tool was used to evaluate the risk of bias summary figure for the 11 RCTs [24].

### 2.3. Data Analysis

We used Python (version 3.7, Python Software Foundation, Python Language Reference, London, UK) to conduct an Apriori algorithm and plot charts [25].  Table 1 shows the summaries of the selected studies from which we extracted and analyzed the acupoint frequencies. In this study, the Apriori algorithm-based association rule analysis and plotting were processed using Python. Using Apriori, we can find hidden relationships in the data. {A} ≥ {B} is defined as the association rule with B when A occurs. We used conditional probability to calculate each acupoint's support, which mathematically is the number of events occurring over the total number. We disregarded acupoints with support of less than 30%.

(1) $$\begin{array}{c} \text{Support}\left(A\right)=P\left(A\right)=\frac{\text{frequency of }A}{\text{total number of point }s}. \end{array}$$

Next, we considered the data confidence and lift. The explicit formulas for confidence and lift are as follows:

  confidence (A ⟶ B) = P(B|A) = P(A ∩ B)/P(A)
  lift (A ⟶ B) = confidence (A ⟶ B)/P(B) = P(A ∩ B)/(P(A) ∗ P(B))

The confidence is A's probability when B occurs. Lift means that A and B have a relative association. That is, if A and B are independent, lift (A ⟶ B) = 1, since P(A ∩ B) = (P(A) ∗ P(B)). If lift (A ⟶ B) = 0, A and B are mutually exclusive events. If the lift is higher than 2, the two objects are highly correlated. We chose acupoints with confidence values higher than 80%, as values lower than this would involve too much data to study, and anything higher involve too little data. We recursively took it to layer three and drew it as a chart.

## 3. Results

### 3.1. Study Characteristics and RoB Assessment

The acupuncture treatment for simple obesity used an average of six acupoints, and the mean period of therapy was 35 days. In this meta-analysis, acupuncture therapy showed statistically significant changes in BMI, BFM, WC, and HC compared to the sham acupuncture group; however, there was no statistically significant difference between the two groups in terms of body weight (BW).

Table 1 summarizes the methodological quality of the retrieved RCTs. Supplementary Table 1 presents the detailed modified Jadad scale scoring assessment [12]. The overall quality of the 11 RCTs was high, with an average Jada score of 5.27. All studies provided a detailed description of their randomized and randomization-hidden protocols and used single-blind designs combined with sham acupuncture treatments. Only three studies elucidated the reasons for patient withdrawals and exits. The RoB summary figure of the included RCTs is provided in Supplementary Figure 1.

### 3.2. Distribution of the Acupoints

**Figure 1:**
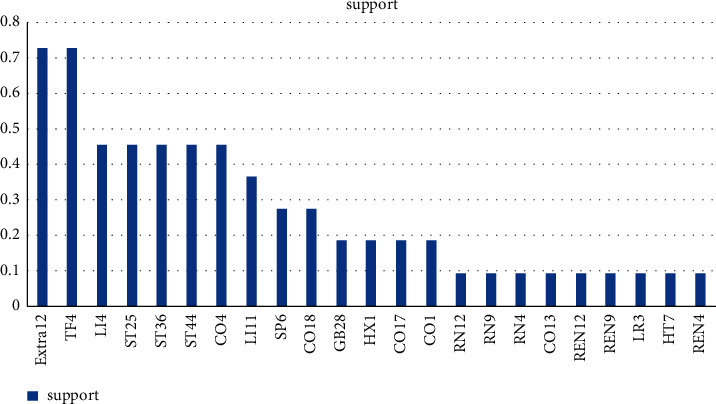
Frequency distribution of acupoints used in the 11 RCTs included in the meta-analysis.

We identified 23 acupoints from the 11 RCTs retrieved from the meta-analysis.  Figure 1 shows the frequency distribution of the acupoints. The top 10 most frequently selected acupoints for simple obesity treatment were Extra12, TF4, LI4, ST25, ST36, ST44, CO4, LI11, SP6, and CO18.

### 3.3. Apriori Algorithm-Based Association Rule Analysis for Item Sets of Acupoint Combinations

**Figure 2:**
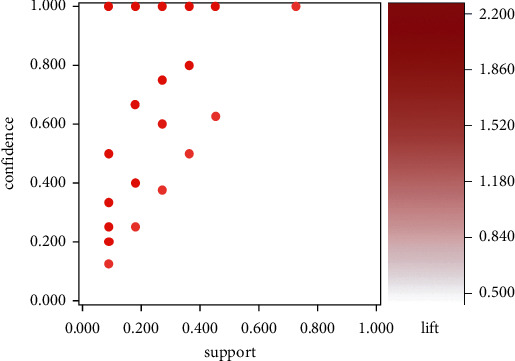
Scatter plot for the 1118 association rules obtained in the 11 RCTs included in the meta-analysis.

We investigated 1,118 association rules based on the acupoints data (Table 1) and deleted those with confidence values lower than 80%. The number 1,118 was calculated by the sum of choosing 2 and 3 out of the 23 identified acupoints and deleting the combinations not in the table. The association rules are shown in Figure 2. The chart shows the acupoints with greater than 80% support. Acupoints Extra12 and TF4 appeared most frequently in the data. We used this to determine the confidence and lift. The top 10 association rules for the selected acupoints are listed in Table 2. We plotted this in a three-dimensional chart (Figure 2).

**Figure 3:**
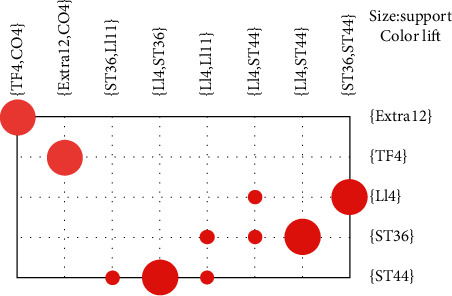
Grouping matrix for the 10 association rules obtained in the 11 RCTs included in the meta-analysis.

We can see the scatter in space. For the grouped item sets, we used graph-based visualization according to color or size. The features were visually presented based on a grouped matrix of 10 association rules (Figure 3). In Section 2.3, we demonstrate that a higher lift indicates a stronger association. If the support and lift values are larger, the circle size will be bigger and red. Otherwise, it will be smaller and lighter. In Table 2 we see that {LI4, ST36}, {LI4, ST44}, and {ST36, ST44} have high support and lift. This association rules were consistent for {LI4, ST36} ≥ {ST44}, {LI4, ST44} ≥ {ST36}, and {ST36, ST44} ≥ { LI4}.

## 4. Discussion

**Figure 4:**
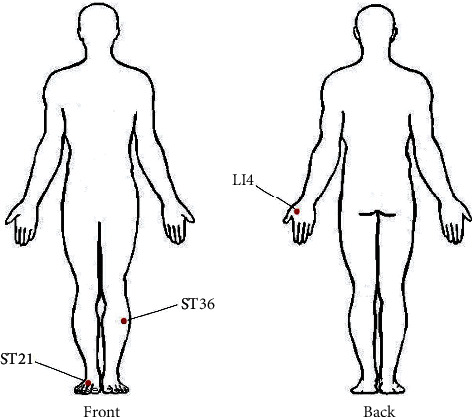
Location of core acupoints in the treatment of patients with obesity.

Our results indicated that LI4, ST36, and ST44; LI4, ST44, and ST36; and ST36, ST44, and LI4 were the core acupoint combinations for treating patients with obesity (Figure 4). Notably, a meta-analysis of acupuncture therapy for simple obesity revealed that the acupoint combination played a significant role in reducing BMI, BFN, WC, and HC. The utility of evidence-based strategies for selecting acupoints for further treatment can be ascertained by their efficacy. This is the first report on potential core acupoint combinations for simple obesity therapy based on RCTs.

Although a few similar studies to the one presented here exist, the data sources of acupuncture for simple obesity that were used had lower levels of evidence, including non-RCTs, case reports, and case series [22, 26, 27]. There are differences in the combinations of acupoints that were reported by clinical trials with different levels of evidence for simple obesity. The core acupoint combination we reported here is different from other studies. For example, the core acupoint combination summarized by Jiang et al. was RN12 and ST36 [27], while when Deng et al. used acupoint catgut embedding, their acupoint combination was reported as RN12 and ST25 [22]. Our study focused on acupuncture for simple obesity, which is different from other studies that have studied acupuncture for secondary obesity, such as diabetes mellitus or hyperlipidemia complicated with obesity [21, 28]. The core acupoint combination summarized here for the treatment of nonsimple obesity and simple obesity is different from those reported in other studies. For instance, the core acupoint combination reported by Sun et al. for treating type 2 diabetes mellitus complicated with obesity was RN12, SP6, and SP9 [21]. In contrast, Wang et al.‘s core acupoint combination for hyperlipidemia complicated with obesity was SP3, ST36, and ST40 [28]. Although our core acupoint combination is different from other studies, ST36 is an acupoint that was identified both by us and by Jiang and Wang [27, 28].

Core acupoint combinations are useful for patients with simple obesity. There are some possible mechanisms as to how acupuncture helps with obesity, including regulating lipid metabolism, modulating inflammatory responses, suppressing appetite, and promoting white adipose tissue (WAT) browning [13]. A C57/B6 mouse model study demonstrated that EA at ST36 could reduce BW by decreasing WAT weight and increasing TRPV1 levels, which regulate lipids [29]. Current studies have confirmed that obesity is related to inflammation and that levels of the proinflammatory cytokines IL-6 and TNF-*α* increase in individuals with obesity [30, 31]. In obese rat models, electroacupuncture at CV12/CV4 can significantly reduce adiponectin and increase serum leptin [32]. It can also increase the levels of the anti-inflammatory factor IL-10 and reduce levels of the proinflammatory factor TNF-*α* [33]. Liu et al. reported that EA at ST36 and ST44 could enhance the excitability of the ventromedial hypothalamic nucleus (VMH) to reduce food intake in obese rats [34]. In addition, in an obese rat model, Qiang et al. found that acupuncture on ST44 and ST36 could decrease adipocyte size and prompt lipolysis in WAT by inducing the expression of uncoupling protein-1 (UCP-1) to promote WAT browning [35].

In clinical disease treatment, acupuncture often uses multiple acupoints at the same time. The therapeutic effect of an acupoint combination is usually better than that of a single acupoint [17, 18]. Animal experiments have shown the same results [36]. Zhang et al. conducted an RCT with hypertension patients; in their study, resting-state fMRI scans indicated that the acupoint combination group using LR3 and KI3 promoted brain activation over a greater area than when using a single acupoint (LR3 or KI3) group [37]. A crossover test using BL21 and CV12 to treat healthy subjects under a water load condition showed lower electrogastrogram (EGG) amplitudes and more new brain region activation than when using a single acupoint (BL21 or CV12) group [38]. Consequently, some evidence suggests that the synergistic pharmacological effect of acupuncture on multiple acupoint combinations is better than that of a single acupoint. The core acupoint combinations found in our article can improve simple obesity by increasing the levels of serum insulin and C peptide and by remodeling WAT to brown adipose tissue (BAT), which has been supported by many modern pharmacological studies [35, 39, 40] (Table 3).

### 4.1. Limitations

There are a few limitations to our study. First, publication bias may occur because there were only 11 trials published in English. None of the studies were from mainland China due to the inclusion criteria. Second, this meta-analysis only included 11 RCTs and a total of 643 patients. Third, the mean therapy duration was 35 days, and only one of the RCTs included had a careful follow-up. Finally, the mechanisms involved in acupoint combinations for treating simple obesity are unclear. Thus, further large-scale, high-quality RCTs are required for a more thorough assessment.

## 5. Conclusions

The acupoint combination of LI4, ST36, and ST44 is the core acupoint combination for simple obesity treatment that could significantly improve BMI, BFN, WC, and HC, as supported by modern pharmacological evidence. However, further rigorous clinical trials are warranted.

## Figures and Tables

**Table 1:** Summary of selected studies.

Study (year)	Study design	Inclusion criteria (kg/m^2^)	Acupoints	Jadad score
Tuğrul Cabioğlu and Ergene (2005) [41]	RCT	BMI > 30	Extra12, TF4, LI 4, LI11, ST25, ST36, ST44	5
Cabioğlu and Ergene (2006) [42]	RCT	BMI > 30	Extra12, TF4, LI 4, LI11, ST25, ST36, ST44	5
Cabioğlu et al. (2008) [43]	RCT	BMI > 30	Extra12, TF4, LI 4, LI11, ST25, ST36, ST44	5
Hsu et al. (2009) [44]	RCT	BMI > 27	TF4, CO4, Extra12, CO18	6
Abdi et al. (2012) [45]	RCT	BMI > 30	TF4, CO4, Extra12, CO1, HX1, CO17	5
Güçel et al. (2012) [46]	RCT	BMI > 30	LI4, HT7, ST36, ST44, SP6	6
Lien et al. (2012) [47]	RCT	BMI > 27	TF4, CO4, Extra12, CO18	6
Darbandi et al. (2013) [48]	RCT	BMI > 25	ST25, GB28, RN12, RN9, RN4, SP6	5
Yeo et al. (2014) [49]	RCT	BMI > 23	TF4, CO4, CO13, Extra12, CO18	5
Darbandi et al. (2014) [50]	RCT	BMI > 30–40	ST25, GB28, REN12, REN9, REN4, SP6, TF4, CO4, Extra12, CO1, HX1, CO17	5
Fogarty et al. (2015) [51]	RCT	BMI > 25	LI4, LI11, ST36, ST44, LR3	5

RCT = randomized controlled trial; BMI = body mass index.

**Table 2:** Apriori algorithm-based association rules for acupoints used for obesity treatment.

No.	Association rules	Support	Confidence	Expected confidence	Lift
1	{Extra12} ≥ {TF4}	0.727272	1.000000	0.727272	1.37500
2	{TF4} ≥ {Extra12}	0.727272	1.000000	0.727272	1.37500
3	{CO4} ≥ {Extra12}	0.454545	1.000000	0.727272	1.37500
4	{CO4} ≥ {TF4}	0.454545	1.000000	0.727272	1.37500
5	{LI4} ≥ {ST36}	0.454545	1.000000	0.454545	2.20000
6	{ST36} ≥ {LI4}	0.454545	1.000000	0.454545	2.20000
7	{LI4} ≥ {ST44}	0.454545	1.000000	0.454545	2.20000
8	{ST44} ≥ {LI4}	0.454545	1.000000	0.454545	2.20000
9	{ST36} ≥ {ST44}	0.454545	1.000000	0.454545	2.20000
10	{ST44} ≥ {ST36}	0.454545	1.000000	0.454545	2.20000
11	{ST25} ≥ {Extra12}	0.363636	0.800000	0.909090	1.10000
12	{ST25} ≥ {TF4}	0.363636	0.800000	0.909090	1.10000
13	{LI4} ≥ {LI11}	0.363636	0.800000	0.454545	2.20000
14	{LI11} ≥ {LI4}	0.363636	1.000000	0.454545	2.20000
15	{LI11} ≥ {ST36}	0.363636	1.000000	0.454545	2.20000
16	{ST36} ≥ {LI11}	0.363636	0.800000	0.454545	2.20000
17	{LI11} ≥ {ST44}	0.363636	1.000000	0.454545	2.20000
18	{ST44} ≥ {ST11}	0.363636	0.800000	0.454545	2.20000

**Table 3:** Potential efficacy of the core acupoints for obesity treatment.

Acupoint	Chinese name	English name	Primary meridians	Efficacy
LI4 [39]	Ho-Ku	Connecting valleys	Large intestine	To increase the levels of serum insulin and C peptide
ST36 [35, 39, 40]	Tsu-San-Li	Walking three miles	Stomach	To increase the levels of serum insulin and C peptide; to remodel WAT to BAT
ST44 [35, 39, 40]	Nei-T'ing	Inner court	Stomach	To increase the levels of serum insulin and C peptide; to remodel WAT to BAT

WAT: white adipose tissue; BAT: brown adipose tissue.

## Data Availability

The original data used to support the study findings are included within the article.

## Supplementary Materials

Supplementary Materials Supplementary Table 1: quality scores of the included RCTs. Supplementary Figure 1: risk of bias of the included RCTs.
