# Supplementary material for: Combined Acupoints for the Treatment of Patients with Obesity: An Association Rule Analysis
Source: Evid Based Complement Alternat Med. 2022 Mar 17;2022:7252213. doi: 10.1155/2022/7252213 (PMC8947926; doi:10.1155/2022/7252213)
Supplement: Supplementary Materials — Supplementary Table 1: quality scores of the included RCTs. Supplementary Figure 1: risk of bias of the included RCTs. [file 7252213.f1.zip › 7252213.f1/Supplementary table 1 Quality scores of the included randomised clinical trials.docx]

**Supplementary table 1: Quality scores of the included randomised clinical trials**

| Study (year) | Randomised | Randomisation hidden | Blinding | Withdrawal and exit | Jadad score |
| --- | --- | --- | --- | --- | --- |
| Cabioğlu et al., 2005 [30] | 2 | 1 | 2 | 0 | 5 |
| Cabioğlu et al., 2006 [31] | 2 | 1 | 2 | 0 | 5 |
| Cabioğlu et al., 2008 [32] | 2 | 1 | 2 | 0 | 5 |
| Hsu et al., 2009 [33] | 2 | 1 | 2 | 1 | 6 |
| Abdi et al., 2012 [34] | 2 | 1 | 2 | 1 | 6 |
| Güçel et al., 2012 [35] | 2 | 1 | 2 | 0 | 5 |
| Lien et al., 2012 [36] | 2 | 1 | 2 | 1 | 6 |
| Darbandi et al., 2013 [37] | 2 | 1 | 2 | 0 | 5 |
| Yeo et al., 2014 [38] | 2 | 1 | 2 | 0 | 5 |
| Darbandi et al., 2014 [39] | 2 | 1 | 2 | 0 | 5 |
| Fogarty et al., 2015 [40] | 2 | 1 | 2 | 0 | 5 |
