# Supplementary figures and images for: Combined Acupoints for the Treatment of Patients with Obesity: An Association Rule Analysis
Source: Evid Based Complement Alternat Med. 2022 Mar 17;2022:7252213. doi: 10.1155/2022/7252213 (PMC8947926; doi:10.1155/2022/7252213)

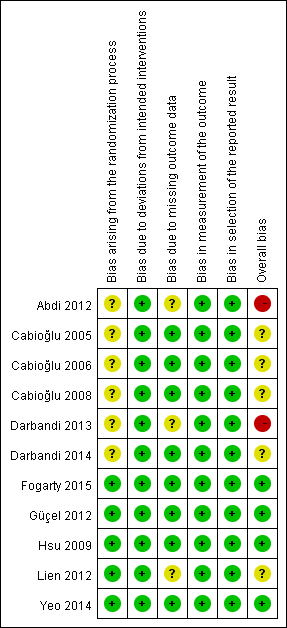

Supplement: Supplementary Materials — Supplementary Table 1: quality scores of the included RCTs. Supplementary Figure 1: risk of bias of the included RCTs. [file 7252213.f1.zip › 7252213.f1/Supplymental figure 1.docx]
